# A non-hepatotropic parasite infection increases mortality in the acetaminophen-induced acute liver failure murine model: possible roles for IL-5 and IL-6

**DOI:** 10.1590/0074-02760160311

**Published:** 2016-10-31

**Authors:** Marco A De León-Nava, Carolina Álvarez-Delgado, Luis Donis-Maturano, Joselin Hernández-Ruiz, Aaron N Manjarrez-Reyna, Edgar Cruz-Avilés, Sonia Leon-Cabrera, Jorge Morales-Montor, José M Fragoso, Galileo Escobedo

**Affiliations:** 1 Centro de Investigación Científica y de Educación Superior de Ensenada, Departamento de Innovación Biomédica, Baja California, México; 2 Universidad Nacional Autónoma de México, Facultad de Medicina, Unidad de Investigación en Medicina Experimental, Hospital General de México Dr Eduardo Liceaga, Laboratorio de Hígado, Páncreas y Motilidad, Ciudad de México, México; 3 Universidad Nacional Autónoma de México, Facultad de Estudios Superiores-Iztacala, Unidad de Biomedicina, Carrera de Médico Cirujano, Los Reyes Iztacala, México; 4 Universidad Nacional Autónoma de México, Instituto de Investigaciones Biomédicas, Departamento de Inmunología, Ciudad de México, México; 5 Instituto Nacional de Cardiología Ignacio Chávez, Departamento de Biología Molecular, Ciudad de México, México

**Keywords:** acute liver failure, acetaminophen, parasite infection, Taenia crassiceps, liver disease, interleukin

## Abstract

We evaluated the effects of a non-hepatotropic parasite infection (*Taenia crassiceps*) on the outcome of acetaminophen-induced acute liver failure in mice. Uninfected and *T. crassiceps* infected mice orally received either 300 mg/kg acetaminophen or water as vehicle (n = 5 per group). Survival analysis, hepatocyte necrosis, alanine aminotransferase (ALT) levels, CYP2E1 protein, interleukin (IL-) 5, and IL-6 were assessed for all groups. All infected mice died within 16 h after exposure to acetaminophen (Tc+APAP group), whereas only one-third of uninfected animals exposed to acetaminophen (APAP group) died. Uninfected (Control group) and infected (Tc group) mice that received the vehicle showed no liver damage. Tc+APAP mice exhibited massive liver necrosis characterised by marked balloning degeneration of hepatocytes and higher serum ALT compared to Control, Tc, and APAP animals. Liver tissue from Tc+APAP mice also displayed increased expression of CYP2E1 protein and higher mRNA and protein levels of IL-5 and IL-6 compared to the other groups. These findings suggest that non-hepatotropic parasite infections may increase mortality following acute liver failure by promoting hepatocyte necrosis via IL-5 and IL-6-dependent CYP2E1 overproduction. This study identifies new potential risk factors associated with severe acute liver failure in patients.

The liver carries out more than 500 functions including regulation of carbohydrate and lipid metabolism, protein synthesis, immune response orchestration, and detoxification processes (Bhatia et al. 2014). Pathological alterations of the liver are considered serious and expensive public health problems, with high mortality rates when relating to either chronic disease or liver failure (Kandiah et al. 2016). Liver failure can be classified into four categories: acute, sub-acute, acute-on-chronic, and chronic liver failure (Bernal et al. 2015). Acute liver failure is the clinical manifestation of sudden and severe hepatic damage in patients having no pre-existing liver disease, and it is frequently related to acetaminophen (APAP) overdose (Chalhoub et al. 2014). Acute liver failure is characterised by massive necrosis of hepatocytes, a sudden increase in alanine aminotransferase (ALT) levels, rapid loss of hepatic function, jaundice, encephalopathy, cerebral edema, multi-organ failure, and extremely high mortality rates ranging from 65-80% (Stravitz & Kramer 2009). Although improved clinical management of acute liver failure has been achieved in the last 20 years, there is still little known regarding the risk factors that increase mortality associated with hepatic disorders.

Acute liver failure is a highly prevalent condition among human populations (Ichai & Samuel 2011). Likewise, parasite infections are common in developing countries, and they even constitute an emerging health problem in developed nations (Chomicz et al. 2016). It is therefore likely that both of these conditions exhibit comorbidity in thousands of people worldwide. In fact, a growing body of evidence has suggested a relationship between parasite infections and the occurrence of liver pathologies. A previous study reported hepatomegaly and altered albumin synthesis in patients with visceral leishmaniasis (Ural et al. 2015). Additionally, patients with fatal falciparum malaria displayed hepatomegaly, jaundice, and ALT elevation (Prommano et al. 2005). An increased number of eosinophils - a common symptom associated with parasite infections - was also observed in the livers of these malaria patients (dos Santos et al. 2009). IL-5 and IL-6 are cytokines with prominent roles during parasite infections, and they have been shown to increase the severity of experimentally induced hepatitis (Louis et al. 2002, dos Santos et al. 2009). Nevertheless, the vast majority of reports have only described the relationship between hepatotropic parasites and liver damage. It is still unknown whether non-hepatotropic parasite infections increase liver damage and mortality during an episode of acute liver failure.

To this end, we examined the effects of a non-hepatotropic parasite infection on the outcome of acute liver failure by inducing an acetaminophen overdose in *Taenia crassiceps*-infected mice. We also explored the potential molecular mechanisms underpinning the observed pathologies.

## MATERIALS AND METHODS

*Mice* - Pathogen-free female BALB/c mice (6-8 weeks old) were obtained from the breeding facilities of the School of Medicine of the National Autonomous University of Mexico. Animals were fed Purine Diet 5015 (Purine, St. Louis, MO, USA) and water *ad libitum*. All experimental procedures were approved by the Ethical Committees of the General Hospital of Mexico and the School of Medicine of the National Autonomous University of Mexico, according to the University Animal Care and Use Committee.

*T. crassiceps inoculation* - *T. crassiceps* larvae were kindly donated by Dr J Morales-Montor of the Biomedical Research Institute of the National Autonomous University of Mexico. Briefly, *T. crassiceps* metacestodes were obtained from a female BALB/c AnN mouse and placed in tubes containing sterile 1x phosphate buffered saline (PBS) supplemented with 100 U/mL penicillin-streptomycin-fungizone (Gibco, Grand Island). Parasites were washed twice with non-supplemented sterile 1x PBS and centrifuged for 10 min at 290 *g* at 4ºC. The supernatant was discarded, and *T. crassiceps* larvae were then resuspended in fresh sterile 1x PBS. Ten viable non-budding *T. crassiceps* larvae approximately 2 mm in diameter were selected for inoculation into the peritoneal cavity of different female mice using a 21 G x 32 mm needle (DL Medica, Mexico). Non-infected control mice were injected with 400 mL sterile 1x PBS instead.

*Acetaminophen overdose and experimental groups* - Eight weeks after infection, both non-infected (n = 10) and infected (n = 10) female mice were subjected to the following treatments: (a) five intraperitoneally inoculated with 400 mL sterile 1x PBS orally received water as a vehicle control (Control group); (b) five animals intraperitoneally inoculated with 400 mL sterile 1x PBS received a single oral dose of 300 mg/kg acetaminophen (APAP group); (c) five animals intraperitoneally inoculated with ten *T. crassiceps* larvae orally received water (Tc group); (d) five animals intraperitoneally inoculated with ten *T. crassiceps* larvae received a single oral dose of 300 mg/kg acetaminophen (Tc+APAP group). The acetaminophen dose was chosen based on previous reports (Mohar et al. 2014). In one set of experiments, all groups were followed for 24 h and mortality rates were recorded (n = 5 mice per group). In a second set of experiments, all groups were euthanised 12 h after treatment with acetaminophen or the vehicle using a CO_2_-saturated chamber, and liver and blood samples were collected immediately (n = 5 mice per group). All sets of experiments were independently repeated twice using five animals per group, as described above.

*Liver histology* - Liver samples were collected from all experimental groups and fixed/stored in 4% paraformaldehyde (JT Baker, Mexico) for two weeks. Briefly, tissues were washed twice with 1x PBS (Sigma-Aldrich, USA) and dehydrated with serial incubations in 70%, 80%, 95%, and 100% ethanol (JT Baker, Mexico) for 15 min each. Tissues were placed into xylene (JT Baker, Mexico) for 30 min. They were washed with 1x PBS and equilibrated in liquid paraffin at 60ºC for 30 min. Liquid paraffin was replaced with fresh 60ºC paraffin, and samples were oriented in embedding blocks. Once the blocks were solidified after 24 h at room temperature, liver tissue was sectioned transversely at 4 mM using a microtome (Microtome Olympus Cut 4060, USA). Liver sections were stained with haematoxylin and eosin to assess hepatocyte necrosis and immune infiltration. Microphotographs were acquired at 40x and 100x magnification using a Nikon Microphot-FXA microscope coupled to a Nikon Digital Camera DXM1200F. Hepatocyte necrosis was estimated by quantifying the number of ballooned hepatocytes in 10 high-powered image fields for each mouse.

*ALT serum levels* - Blood samples were collected from all experimental groups and centrifuged at 454 *g* for 30 min. Serum samples were isolated and stored at -86ºC until use. Serum levels of ALT were determined by a conventional colorimetric/fluorometric assay that results in pyruvate formation proportional to ALT activity (Sigma-Aldrich, Mexico).

*CYP2E1 detection by western blot* - Hepatic tissue from each experimental animal was disrupted in a protein extraction buffer containing a protease inhibitor cocktail (Calbiochem, Darmstadt, Germany) in 500 mM Tris-HCl (1 mL/0.1 g tissue). After 15 min of centrifugation at 20800 *g*, the supernatant was recovered. Protein was quantified by absorbance at 595 nm using the Bradford and Lowry methods (Seevaratnam et al. 2009). In each case, 20 mg of total protein extract was boiled in Laemmli sample buffer, separated by SDS-PAGE (10% acrylamide), and transferred to PVDF membranes. The PVDF membranes were blocked overnight in 1x PBS supplemented with 0.2% Tween 20 and 1% BSA. Membranes were washed five times in 1x PBS-Tween 20 and separately incubated for 1 h at room temperature with rabbit anti-mouse CYP2E1 antibody (Santa Cruz Biotech, CA, USA). Membranes were then washed three times in 1x PBS-Tween 20 and incubated for 1 h with goat anti-rabbit IgG-HRP antibody (Santa Cruz Biotech, CA, USA). Protein bands were visualised by the peroxidase-diaminobenzidine reaction according to the manufacturer’s instructions (Sigma-Aldrich, Mexico). CYP2E1 protein bands were quantified by optical density (OD) analysis using a-TUBULIN as a control.

*Total RNA isolation* - Liver samples were placed in TRIzol reagent (Invitrogen, USA) and stored at -70ºC until use. Briefly, hepatic tissue was disrupted in TRIzol reagent (1 mL/0.1 g tissue) at 4ºC. Subsequently, 0.2 mL cold chloroform was added to each sample for every 1 mL of TRIzol used. After a 10 min incubation at 4ºC, samples were centrifuged at 20800 *g* for 15 min at 4ºC. The aqueous phase of each sample was recovered and separately placed into 1.6 mL Eppendorf tubes. Total RNA was then precipitated with isopropyl alcohol overnight at 4ºC. After this, RNA samples were centrifuged at 20800 *g* for 15 min at 4ºC and the supernatant was discarded. The RNA pellet was washed twice with 100% ethanol and dissolved in RNAse-free water treated with DEPC (Sigma-Aldrich, USA). The RNA concentration was determined by absorbance at 260/280 nm, and purity was verified by electrophoresis on a 1.0% denaturing agarose gel (Promega, Uniparts, Mexico) in the presence of 2.2 M formaldehyde.

*Quantification of IL-5 and IL-6 expression by reverse transcriptase polymerase chain reaction (RT-PCR)* - To quantify the mRNA expression of *IL-5* and *IL-6*, total RNA samples from hepatic tissues were reverse-transcribed using the M-MLV Retrotranscriptase system and oligo(dT) primer (Invitrogen, USA). The resulting cDNAs were employed for semi-quantitative PCR using TaqDNA polymerase (Biotecnologías Universitarias, UNAM, México) and gene-specific primers for *IL-5* (forward: 5ˊ-ACATGCTGGGCCTTACTTCT-3ˊ; reverse: 5ˊ-TGGAGTAAACTGGGGGAGGC-3ˊ; product length: 151 bp) and *IL-6* (forward: 5ˊ-GCCTTCTTGGGACTGATGCT-3ˊ; reverse: 5ˊ-CTGCAAGTGCATCATCGTTGT-3ˊ; product length: 217 bp). 18S-ribosomal RNA (forward: 5ˊ-CGCGGTTCTATTTTGTTGGT-3ˊ; reverse: 5ˊ-AGTCGGCATCGTTTATGGTC-3ˊ; product length: 219 bp) was used as a constitutively expressed housekeeping gene for normalisation. Briefly, the 25-µL PCR reaction contained 2 µL cDNA, 5 µL 10x PCR-buffer (Perkin-Elmer, USA), 1 mM MgCl_2_, 0.2 mM of each dNTP, 0.05 µM of each gene-specific primer, 0.5 µL TaqDNA polymerase (Biotecnologías Universitarias, Mexico), and DNAse-free water. After an initial denaturation step at 94ºC for 5 min, the following PCR program was performed for either 28 or 30 cycles (depending on the primer sequence): denaturation at 95ºC for 30 s, annealing at temperatures ranging from 58ºC to 62ºC (depending on the primer sequence) for 30 s, and extension at 72ºC for 45 s. A final extension step was completed at 72ºC for 5 min for each experiment. The 25-µL PCR reactions were then separated by electrophoresis on a 2% agarose gel (Promega, Uniparts, Mexico). The corresponding bands were visualised by staining with ethidium bromide and compared to a 100 bp ladder as a molecular weight marker (Fermentas, Mexico). Relative expression of each amplified gene was determined by OD analysis and normalised to the expression of 18S-ribosomal RNA.

*IL-5 and IL-6 protein quantification by enzyme-linked immunosorbent assay (ELISA)* - Liver samples were collected and stored in protein extraction buffer at -70ºC until use. Subsequently, 2 g of liver tissue from each animal was individually disrupted in a protein extraction buffer (Calbiochem, Darmstadt, Germany). After centrifugation at 20800 *g* for 15 min at 4ºC, the supernatant was recovered. Protein concentrations were determined by absorbance at 595 nm using the Bradford and Lowry methods (Seevaratnam et al. 2009). We used 1 mg total protein to determine IL-5 and IL-6 concentrations in triplicate using the ELISA according to the manufacturer’s instructions (Peprotech, Mexico). All ELISA measurements were performed at the same time to avoid procedural variations.

**Fig. 3:**
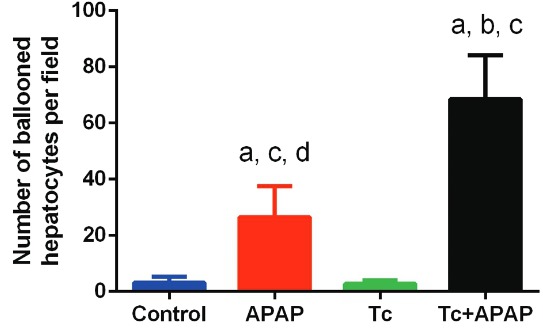
: number of ballooned hepatocytes in *Taenia crassiceps*-infected mice following acetaminophen (APAP) overdose. Hepatocyte necrosis was estimated by quantifying the number of ballooned hepatocytes in 10 high-powered imaging fields from each mouse. Acetaminophen exposure induced a 9-fold increase in the amount of necrotic hepatocytes in APAP mice compared to control and Tc animals. Acetaminophen exposure tripled the number of ballooned hepatocytes following non-hepatotropic parasite infection. Experimental sets were repeated twice under independent conditions using five animals per group in each individual experiment. Data are presented as the mean ± standard deviation. (a) Significant differences compared to the control group; (b) significant differences compared to the APAP group; (c) significant differences compared to the Tc group; (d) significant differences compared to the Tc+APAP group.

**Fig. 4:**
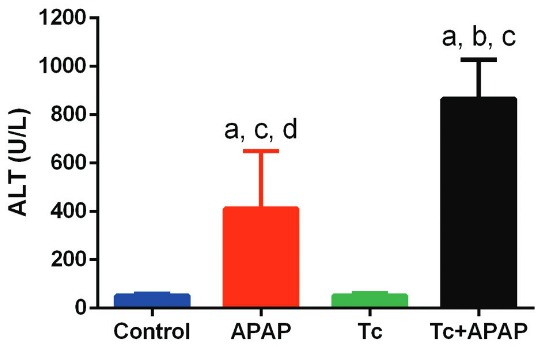
: quantification of serum alanine aminotransferase (ALT) levels in *Taenia crassiceps*-infected mice following acetaminophen (APAP) overdose. Tc+APAP animals exhibited the most significant elevation in ALT serum levels compared to control, Tc, and APAP mice. Serum concentrations of ALT were higher in APAP animals than in control and Tc mice. ALT serum levels fell within normal ranges in control and Tc animals. Experimental sets were repeated twice under independent conditions using five animals per group in each individual experiment. Data are presented as the mean ± standard deviation. (a) Significant differences compared to the control group; (b) significant differences compared to the APAP group; (c) significant differences compared to the Tc group; (d) significant differences compared to the Tc+APAP group.

**Fig. 5:**
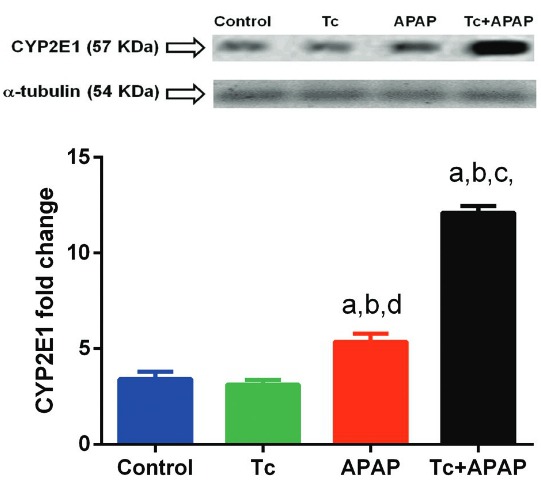
: quantification of CYP2E1 protein levels in liver tissue of *Taenia crassiceps*-infected mice following acetaminophen (APAP) overdose. Native CYP2E1 protein showed a significant 4-fold increase in liver tissue of Tc+APAP mice compared to that in control and Tc animals. CYP2E1 protein levels were 2.3-fold higher in liver tissue of Tc+APAP animals compared to APAP mice, although CYP2E1 expression was 1.8-fold higher in hepatic specimens of APAP animals compared to that in control and Tc mice. A representative acrylamide/bis-acrylamide gel is shown (upper panel). CYP2E1 protein was quantified by optical density (OD) analysis using a-TUBULIN as a control (bottom panel). Experimental sets were repeated twice under independent conditions using five animals per group in each individual experiment. Data are presented as the mean ± standard deviation. (a) Significant differences compared to the control group; (b) significant differences compared to the APAP group; (c) significant differences compared to the Tc group; (d) significant differences compared to the Tc+APAP group.

**Fig. 6:**
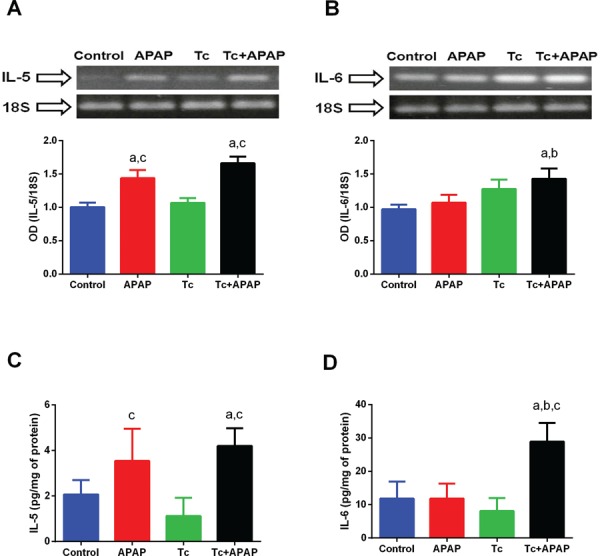
: quantification of mRNA and protein levels of IL-5 and IL-6 in liver tissue of *Taenia crassiceps*-infected mice following acetaminophen (APAP) overdose. (A) Hepatic expression of *IL-5* was significantly higher in APAP and Tc+APAP animals compared to that in control and Tc mice. No significant differences were detected between APAP and Tc+APAP animals; (B) hepatic expression of *IL-6* was significantly higher in Tc+APAP mice compared to that in control, APAP, and Tc animals. Representative agarose gels are shown (upper panels). *IL-5* and *IL-6* gel bands were quantified by optical density analysis using 18S-ribosomal RNA as a constitutively expressed control gene (bottom panels); (C) Tc+APAP and APAP mice displayed higher hepatic protein levels of IL-5 compared to control and Tc animals, but no significant difference when compared to each other (Tc+APAP versus APAP mice); (D) IL-6 protein levels were higher in the livers of Tc+APAP animals compared to those in the other groups. Cytokine quantification was calculated per mg of total liver protein by ELISA. Experimental sets were repeated twice under independent conditions using five animals per group in each individual experiment. Data are presented as the mean ± standard deviation. (a) Significant differences compared to the control group; (b) significant differences compared to the APAP group; (c) significant differences compared to the Tc group.

*Statistics* - Survival analysis was performed using Kaplan-Meier curves followed by the Log-rank Mantel-Cox test for comparison among curves. In Figs 3-6, data are presented as mean ± standard deviation (SD), and analysed using the Shapiro-Wilk test for determination of normality. Data were analysed by one-way ANOVA followed by the post-hoc Tukey test using the GraphPad Prism 5 software. Both experiments performed at 12 h and 24 h were repeated twice under independent conditions using five animals per group in each individual replicate. Differences were considered significant if p < 0.05.

## RESULTS

**Fig. 1:**
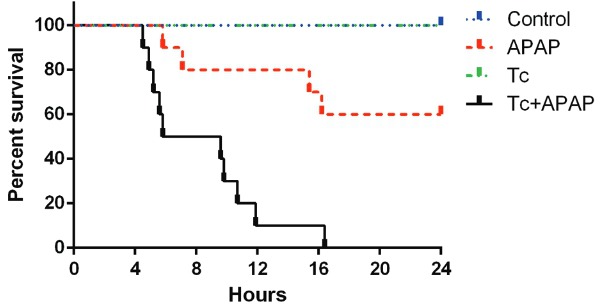
: Kaplan-Meier curves showing percent survival of *Taenia crassiceps*-infected mice following an overdose of acetaminophen. BALB/c AnN female mice were inoculated with either 10 *T. crassiceps* larvae or 1x phosphate buffered saline (PBS). Eight weeks after *T. crassiceps* or 1x PBS inoculation, animals received a single oral dose of 300 mg/kg acetaminophen (APAP) or sterile water as a vehicle control. Survival was significantly lower in Tc+APAP mice compared to that in control, Tc, and APAP animal groups (p < 0.001, p < 0.001, and p = 0.001, respectively). Survival analysis was performed using Kaplan-Meier curves followed by the Log-rank Mantel-Cox test for comparison among curves. Survival rates were obtained from experimental sets repeated twice under independent conditions using five animals per group in each individual experiment. Control: uninfected animals receiving sterile water; APAP: uninfected animals receiving 300 mg/kg acetaminophen; Tc: infected animals receiving sterile water; Tc+APAP: infected animals receiving 300 mg/kg acetaminophen.

*T. crassiceps infection increases mortality in the acetaminophen-induced acute liver failure murine model* - The APAP group showed 15% mortality 12 h after acetaminophen administration (Fig. 1). The maximum mortality rate in this group was observed by 17 h, at which time 35% of the animals had died. In contrast, the Tc+APAP group exhibited 85% mortality during the first 12 h of the experiment, reaching 100% by 16 h (Fig. 1). The control and Tc groups showed 0% mortality throughout the experiment (Fig. 1). Interestingly, the number of peritoneal parasites did not change in response to acetaminophen overdose (Tc+APAP group: 437 ± 129, Tc group: 412 ± 156).

**Fig. 2:**
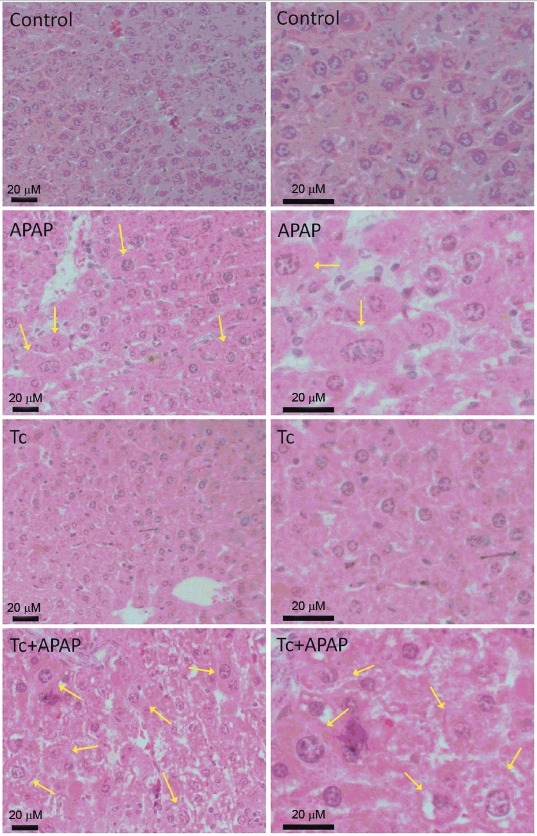
: ballooning degeneration of hepatocytes in *Taenia crassiceps*-infected mice following an overdose of acetaminophen (APAP). Significantly more ballooned hepatocytes were observed in Tc+APAP mice compared to APAP animals. Liver tissue from control and Tc mice did not show ballooning degeneration. Left panels show representative microphotographs at 40x magnification and right panels show the same images enlarged at 100x. Hepatocyte damage can be seen in more detail. Scale bars: 20 mM; yellow arrows: ballooned hepatocytes. Liver samples were collected in experimental sets repeated twice under independent conditions using five animals per group in each individual experiment. Control: uninfected animals receiving sterile water; APAP: uninfected animals receiving 300 mg/kg acetaminophen; Tc: infected animals receiving sterile water; Tc+APAP: infected animals receiving 300 mg/kg acetaminophen.

*T. crassiceps infection is associated with increased hepatocyte necrosis in the acetaminophen-induced acute liver failure murine model* - Ballooning degeneration of hepatocytes is a well-established indicator of hepatic necrosis. Liver histology revealed that Tc+APAP mice displayed higher numbers of ballooned hepatocytes than APAP animals (Fig. 2). Hepatocytes from control and Tc groups did not exhibit ballooning degeneration as that observed in Tc+APAP and APAP animals (Fig. 2). Quantification of the number of ballooned cells confirmed that acetaminophen exposure induced a 9-fold increase in the amount of necrotic hepatocytes in APAP mice (26.5 ± 10.93) when compared to control and Tc animals (3.16 ± 2.13 and 2.83 ± 1.16, respectively). However, acetaminophen exposure tripled the number of ballooned hepatocytes when compared to the parasite-infected Tc+APAP group (68.5 ± 15.6) (Fig. 3). Furthermore, little to no leukocyte infiltration was found in the livers of Tc+APAP and APAP mice. This was similar to the control and Tc animals in which no immune cell infiltration was observed as well.

As expected based upon the amount of ballooned hepatocytes, the Tc+APAP animals exhibited the most significant elevation in ALT serum levels compared to the control, Tc, and APAP groups (Fig. 4). Specifically, Tc+APAP mice showed 2-fold higher values for ALT serum than APAP animals (865.2 ± 162.9 U/L and 411.9 ± 238.4 U/L, respectively) (Fig. 4). In contrast, serum ALT levels were significantly lower in control (51.67 ± 8.87 U/L) and Tc (52.33 ± 10.61 U/L) animals compared to APAP and Tc+APAP mice (Fig. 4). It is worth mentioning that ALT serum concentrations fell within normal ranges for control and Tc animals, which suggested that the presence of the parasites themselves had no impact on liver function.

*T. crassiceps infection increases CYP2E1 expression in the acetaminophen-induced acute liver failure murine model* - In hepatocytes, acetaminophen is metabolised by the cytochrome P450 family 2 subfamily E member 1 (CYP2E1). Native CYP2E1 protein (57 kDa) showed a significant 4-fold increase in the liver tissue of Tc+APAP mice when compared to control and Tc animals (12.1 ± 0.35 versus 3.4 ± 0.38 and 3.1 ± 0.24, respectively) (Fig. 5). CYP2E1 protein levels also exhibited a 2.3-fold increase in the liver tissue of Tc+APAP animals compared to those in APAP mice (12.1 ± 0.35 and 5.3 ± 0.41, respectively), although CYP2E1 production was 1.8-fold higher in hepatic specimens of APAP animals compared to that in control and Tc mice (Fig. 5). No differences in the levels of a-TUBULIN (54 KDa) were observed among the experimental groups, indicating that changes in CYP2E1 expression were not related to variations in protein quantification (Fig. 5, upper panel).

*T. crassiceps infection differentially induces IL-5 and IL-6 expression in the acetaminophen-induced acute liver failure murine model* - The hepatic expression of *IL-5* was significantly higher in APAP and Tc+APAP animals compared to that in control and Tc mice (Fig. 6A). In contrast, no differences in *IL-5* expression levels were detected between the APAP and Tc+APAP animal groups (Fig. 6A). Moreover, mRNA levels of *IL-6* were significantly higher in the liver tissue of Tc+APAP mice compared to those in control and APAP animals (Fig. 6B). Although the hepatic expression of *IL-6* was elevated in Tc mice, this was not a significant increase compared to expression levels in control and APAP animals (Fig. 6B). Consistent with these RT-PCR results, Tc+APAP and APAP mice displayed a 2-fold higher level of hepatic IL-5 protein when compared to control and Tc animals (Fig. 6C). However, no significant differences were observed in Tc+APAP mice versus APAP mice (Fig. 6C). Similarly, the level of IL-6 protein was 3-fold higher in the livers of Tc+APAP animals compared to that in control, Tc, and APAP mice (Fig. 6D). These results cumulatively suggested that IL-5 was mainly produced in response to liver damage, whereas IL-6 played a synergistic role when liver damage occurred in conjunction with a parasite infection.

## DISCUSSION

In this study, we showed that the comorbidity of acetaminophen-induced hepatotoxicity and non-hepatotropic parasite infections increased mortality associated with acute liver failure. Both chronic and acute liver complications are common causes of mortality worldwide (Kandiah et al. 2016). Furthermore, parasite infections are a public health problem in developing countries and they constitute an emerging concern even in developed nations (Fleury et al. 2012, Fabiani & Bruschi 2013, Chomicz et al. 2016). Since liver disease and parasite infections are prevalent conditions in many countries, their comorbidity is highly likely. Therefore, it is of great clinical relevance to examine whether the presence of parasites causes more severe liver injury, poorer prognoses, and/or increased mortality in patients with acute hepatic disease.

In order to study the possible relationship between acute liver failure and parasite infection, we used two established experimental paradigms to induce acute liver injury in parasitised mice. Specifically, we inoculated mice with *T. crassiceps* parasitic larvae and administered an overdose of acetaminophen to cause liver damage (Vargas-Villavicencio et al. 2007, Xie et al. 2015). The acetaminophen-induced acute liver failure in mice resembles many aspects of acetaminophen overdose in humans, including hepatocyte necrosis, ALT serum elevation, and massive liver injury (Mohar et al. 2014, Maes et al. 2016). Likewise, inoculation of *T. crassiceps* larvae into the peritoneal cavities of mice has been useful for studying the interactions between steroid hormones and immune cells in the context of a chronic infection (Morales-Montor et al. 2008). Here, we found that mortality associated with acute liver failure increased by 100% as a result of the comorbidity of a parasitic infection. Despite recent studies, there is very little information concerning the potential complications of having liver disease and a parasite infection at the same time. A few reports have described hepatomegaly, jaundice, hepatic malfunction, and poor prognoses in patients infected with parasites (Prommano et al. 2005, Ural et al. 2015). However, the aforementioned studies only examined clinical cases of parasites that already show an affinity for infiltrating the liver, such as *Plasmodium falciparum* and *Fasciola hepatica* (Nacher et al. 2001, Haseeb et al. 2003). This limitation has made it difficult to elucidate whether non-hepatotropic parasites are capable of directly enhancing liver injury, or whether liver injury is simply the consequence of hepatic decompensation. For this reason, our experiments involved a liver injury in the presence of a non-hepatotropic parasite infection. Specifically, this parasite proliferated in the mouse peritoneal cavity without invading the liver. This strategy demonstrated that non-hepatotropic parasites are indeed able to increase liver damage directly, providing some insights into the molecular mechanisms involved.

Acute liver failure results from necrosis of hepatocytes and a sudden release of ALT into the blood stream. Usually, this involves little to no immune cell infiltration of the liver (Bernal et al. 2015, Yoon et al. 2016). In this study, we found that ballooning degeneration of hepatocytes (a well-established indicator of hepatic necrosis) (Dias et al. 2007) was significantly increased, following simultaneous acetaminophen overdose and *T. crassiceps* infection. Interestingly, levels of serum ALT were higher in parasitised mice that received acetaminophen than in non-parasitised mice that received acetaminophen. In parallel, we observed a mild-to-absent leukocyte infiltration in hepatic specimens of mice that received acetaminophen independent of parasite infection, suggesting that the parasite itself was unable to promote a generalised immune cell response that might exacerbate liver injury. These findings support the idea that the severity of acute liver failure may be related to multiple conditions, such as non-hepatotropic parasite infections. Therefore, it is necessary to further investigate risk factors that worsen the prognoses of patients with acute liver injury.

One possible explanation for the relationship between acute liver injury and non-hepatotropic parasite infections might be an interaction between CYP2E1 and IL-6. CYP2E1 is a member of the cytochrome C superfamily, and it has prominent roles in metabolising a wide variety of substances like acetaminophen (Kessova & Cederbaum 2003). When acetaminophen accumulates, CYP2E1 is activated and it produces N-acetyl-p-benzoquinone imine (NAPQI), a reactive metabolite capable of binding to mitochondrial proteins. This leads to peroxynitrite formation and mitochondrial oxidative stress (Cover et al. 2005). Increased oxidative stress causes the mitochondrial permeability transition pore to open, resulting in a disruption of mitochondrial membrane potential, inhibition of ATP synthesis, mitochondrial dysfunction, DNA fragmentation, and necrosis of hepatocytes (Maes et al. 2016). Similarly, our data demonstrated that acetaminophen overdose increased CYP2E1 production in the livers of APAP mice. We also found that CYP2E1 expression increased following liver injury in parasitised mice. This suggests a role for *T. crassiceps* in modulating CYP2E1 expression, which might promote the formation of NAPQI and induce hepatic necrosis. A seminal study previously demonstrated that *T. crassiceps* increases expression of CYP19A1 (a member of the cytochrome P450 superfamily) in the testicles of parasitised male mice (Morales-Montor et al. 1999). Interestingly, investigations revealed that the parasite achieves this, in part, through upregulation of IL-6 (Morales-Montor et al. 2001). IL-6 expression has been shown to increase in response to *T. crassiceps* infection (Morales-Montor et al. 2001, 2008). Furthermore, IL-6 upregulates the expression of several members of the cytochrome C superfamily by activating distal intragenic enhancers (Zhao et al. 1997). In our study, mRNA and protein levels of IL-6 were elevated in the livers of parasitised mice that experienced an overdose of acetaminophen. Interestingly, the mRNA levels of IL-6 were higher in the livers of infected animals not exposed to the hepatotoxic agent, although this was not statistically significant. Cumulatively, this suggests that *T. crassiceps* upregulates CYP2E1 by inducing IL-6 expression in liver tissue through a possible enzyme-cytokine mechanism that may be enhanced during acute liver injury. However, additional studies in CYP2E1- and IL-6-deficient mice are needed to understand the potential effects of non-hepatotropic parasites on mortality during acute liver failure.

Our study also identified a potential role for IL-5 in the pathology of acetaminophen-induced acute liver failure. IL-5 is a Th2 cytokine that plays pivotal roles in diseases caused by parasitic helminths. IL-5 is also associated with increased liver injury in hepatic disease models, including LPS-induced hepatotoxicity and concanavalin A (ConA)-induced hepatitis (Louis et al. 2002). Furthermore, IL-5 promotes hepatic necrosis in the ConA-induced T cell-mediated hepatitis murine model (Duran et al. 2004). However, to the best of our knowledge, this is the first study reporting an upregulation of IL-5 in the livers of mice exposed to acetaminophen overdose. It is well known that IL-5 increases liver injury by recruiting leukocytes into the hepatic parenchyma (Jaruga et al. 2003). However, acetaminophen-induced acute liver failure is typically characterised by little to no leukocyte infiltration in the hepatic parenchyma, which we likewise observed in this study. Therefore, there are two possible explanations: (a) IL-5 causes liver injury in a leukocyte-independent fashion or (b) IL-5 expression is an indirect consequence of liver damage and has no influence on the pathology. Therefore, we argue that it is of clinical importance for future studies to explore whether IL-5 levels in human liver biopsies or serum samples could be a useful predictor of the prognosis of severe acute liver failure in patients.

In conclusion, our results demonstrate that a non-hepatotropic parasite infection is capable of increasing the mortality of mice following acute liver injury. The underlying mechanism might involve a synergism among CYP2E1, IL-6, and IL-5 that leads to hepatocyte necrosis. Further clinical research is needed to evaluate the potential benefits of using IL-5 and/or IL-6 as possible biomarkers for identifying patients with a higher risk of developing severe acute liver failure.

## References

[B1] Bernal W, Jalan R, Quaglia A, Simpson K, Wendon J, Burroughs A (2015). Acute-on-chronic liver failure. Lancet.

[B2] Bhatia SN, Underhill GH, Zaret KS, Fox IJ (2014). Cell and tissue engineering for liver disease. Sci Transl Med.

[B3] Chalhoub WM, Sliman KD, Arumuganathan M, Lewis JH (2014). Drug-induced liver injury: what was new in 2013?. Expert Opin Drug Metab Toxicol.

[B4] Chomicz L, Conn DB, Szaflik JP, Szostakowska B (2016). Newly emerging parasitic threats for human health: national and
international trends. BioMed Res Int.

[B5] Cover C, Mansouri A, Knight TR, Bajt ML, Lemasters JJ, Pessayre D (2005). Peroxynitrite-induced mitochondrial and endonuclease-mediated nuclear
DNA damage in acetaminophen hepatotoxicity. J Pharmacol Exp Ther.

[B6] Dias LB, Alves VA, Kanamura C, Oikawa RT, Wakamatsu A (2007). Fulminant hepatic failure in northern Brazil: morphological,
immunohistochemical and pathogenic aspects of Labrea hepatitis and yellow
fever. Trans R Soc Trop Med Hyg.

[B7] Santos DC dos, Martinho JMSG, Pacheco-Moreira LF, Araujo CCV de, Caroli-Bottino A, Pannain VL (2009). Eosinophils involved in fulminant hepatic failure are associated with
high interleukin-6 expression and absence of interleukin-5 in liver and peripheral
blood. Liver Int.

[B8] Duran A, Rodríguez A, Martin P, Serrano M, Flores JM, Leitges M (2004). Crosstalk between PKCzeta and the IL4/Stat6 pathway during
T-cell-mediated hepatitis. EMBO J.

[B9] Fabiani S, Bruschi F (2013). Neurocysticercosis in Europe: still a public health concern not only
for imported cases. Acta Trop.

[B10] Fleury A, Sciutto E, Larralde C (2012). Neurocysticercosis is still prevalent in Mexico. Salud Publica Mex.

[B11] Haseeb AN, el-Shazly AM, Arafa MA, Morsy AT (2003). Evaluation of excretory/secretory Fasciola (Fhes) antigen in diagnosis
of human fascioliasis. J Egypt Soc Parasitol.

[B12] Ichai P, Samuel D (2011). Epidemiology of liver failure. Clin Res Hepatol Gastroenterol.

[B13] Jaruga B, Hong F, Sun R, Radaeva S, Gao B (2003). Crucial role of IL-4/STAT6 in T cell-mediated hepatitis: up-regulating
eotaxins and IL-5 and recruiting leukocytes. J Immunol.

[B14] Kandiah PA, Olson JC, Subramanian RM (2016). Emerging strategies for the treatment of patients with acute hepatic
failure. Curr Opin Crit Care.

[B15] Kessova I, Cederbaum AI (2003). CYP2E1: biochemistry, toxicology, regulation and function in
ethanol-induced liver injury. Curr Mol Med.

[B16] Louis H, Le Moine A, Flamand V, Nagy N, Quertinmont E, Paulart F (2002). Critical role of interleukin 5 and eosinophils in concanavalin
A-induced hepatitis in mice. Gastroenterol.

[B17] Maes M, Vinken M, Jaeschke H (2016). Experimental models of hepatotoxicity related to acute liver
failure. Toxicol Appl Pharmacol.

[B18] Mohar I, Stamper BD, Rademacher PM, White CC, Nelson SD, Kavanagh TJ (2014). Acetaminophen-induced liver damage in mice is associated with
gender-specific adduction of peroxiredoxin-6. Redox Biol.

[B19] Morales-Montor J, Baig S, Mitchell R, Deway K, Hallal-Calleros C, Damian RT (2001). Immunoendocrine interactions during chronic cysticercosis determine
male mouse feminization: role of IL-6. J Immunol.

[B20] Morales-Montor J, Escobedo G, Vargas-Villavicencio JA, Larralde C (2008). The neuroimmunoendocrine network in the complex host-parasite
relationship during murine cysticercosis. Curr Top Med Chem.

[B21] Morales-Montor J, Rodríguez-Dorantes M, Cerbon MA (1999). Modified expression of steroid 5 alpha-reductase as well as aromatase,
but not cholesterol side-chain cleavage enzyme, in the reproductive system of male
mice during (Taenia crassiceps) cysticercosis. Parasitol Res.

[B22] Nacher M, Treeprasertsuk S, Singhasivanon P, Silachamroon U, Vannaphan S, Gay F (2001). Association of hepatomegaly and jaundice with acute renal failure but
not with cerebral malaria in severe falciparum malaria in Thailand. Am J Trop Med Hyg.

[B23] Prommano O, Chaisri U, Turner GD, Wilairatana P, Ferguson DJ, Viriyavejakul P (2005). A quantitative ultrastructural study of the liver and the spleen in
fatal falciparum malaria. Southeast Asian J Trop Med Public Health.

[B24] Seevaratnam R, Patel BP, Hamadeh MJ (2009). Comparison of total protein concentration in skeletal muscle as
measured by the Bradford and Lowry assays. J Biochem.

[B25] Stravitz RT, Kramer DJ (2009). Management of acute liver failure. Nat Rev Gastroenterol Hepatol.

[B26] Ural S, Kaptan F, Sezak N, El S, Örmen B, Türker N (2015). Evaluation of clinical and laboratory findings of adult visceral
leishmaniasis cases. Mikrobiyol Bul.

[B27] Vargas-Villavicencio JA, Larralde C, De León-Nava MA, Escobedo G, Morales-Montor J (2007). Tamoxifen treatment induces protection in murine
cysticercosis. J Parasitol.

[B28] Xie J, Liu J, Chen TM, Lan Q, Zhang QY, Liu B (2015). Dihydromyricetin alleviates carbon tetrachloride-induced acute liver
injury via JNK-dependent mechanism in mice. World J Gastroenterol.

[B29] Yoon E, Babar A, Choudhary M, Kutner M, Pyrsopoulos N (2016). Acetaminophen-Induced Hepatotoxicity: a comprehensive
update. Journal Clin Transl Hepatol.

[B30] Zhao Y, Agarwal VR, Mendelson CR, Simpson ER (1997). Transcriptional regulation of CYP19 gene (aromatase) expression in
adipose stromal cells in primary culture. J Steroid Biochem Mol Biol.

